# Phase separation and transcriptional regulation in cancer development

**DOI:** 10.7555/JBR.37.20230214

**Published:** 2024-05-29

**Authors:** Yan Gu, Ke Wei, Jun Wang

**Affiliations:** Department of Thoracic Surgery, Jiangsu Province Hospital, the First Affiliated Hospital of Nanjing Medical University, Nanjing, Jiangsu 210029, China

**Keywords:** phase separation, transcription regulation, cancer, super-enhancer, condensates

## Abstract

Liquid-liquid phase separation, a novel biochemical phenomenon, has been increasingly studied for its medical applications. It underlies the formation of membrane-less organelles and is involved in many cellular and biological processes. During transcriptional regulation, dynamic condensates are formed through interactions between transcriptional elements, such as transcription factors, coactivators, and mediators. Cancer is a disease characterized by uncontrolled cell proliferation, but the precise mechanisms underlying tumorigenesis often remain to be elucidated. Emerging evidence has linked abnormal transcriptional condensates to several diseases, especially cancer, implying that phase separation plays an important role in tumorigenesis. Condensates formed by phase separation may have an effect on gene transcription in tumors. In the present review, we focus on the correlation between phase separation and transcriptional regulation, as well as how this phenomenon contributes to cancer development.

## Introduction

**Figure 1:**
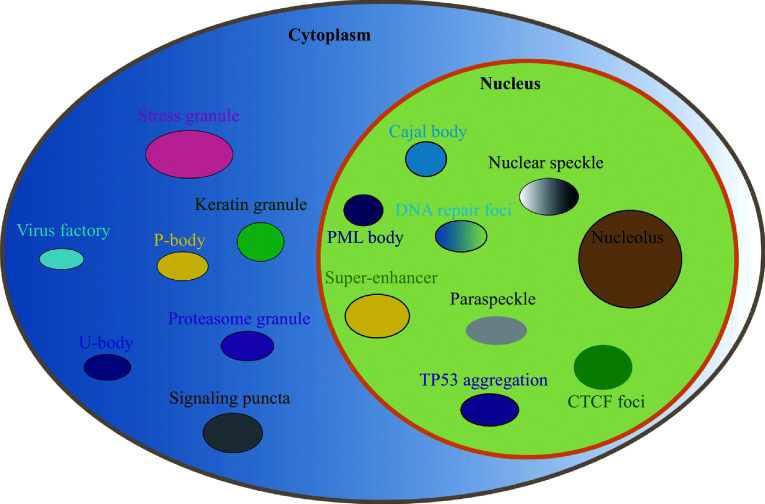
Membrane-less organelles in the cells.

Liquid-liquid phase separation (LLPS) is a physicochemical and thermodynamic process that typically refers to the spontaneous separation of a well-mixed solution of macromolecules, such as proteins or nucleic acids, into two phases (dense and dilute), to achieve the lowest free-energy state^[1]^. The dense phase is morphologically similar to a droplet and exhibits liquid-like properties. It forms a compartment-like structure called condensates that physically separate the interior of the phase from the exterior, enriching some macromolecules within the droplet and excluding others^[2]^. Condensates formed by phase separation have different properties, including dynamic liquid-like, non-dynamic gel-like, and solid amyloid-like properties, and generally consist of protein-protein, protein-RNA, or protein-DNA mixtures^[3]^. To achieve spatial and temporal control of complex biochemical reactions, cells must organize proteins and other macromolecules into subcellular compartments. In addition to conventional membrane-bound organelles, several membrane-less organelles are present within a cell, such as nucleoli, Cajal bodies, and promyelocytic leukemia protein (PML) bodies in the nucleus and stress granules (SGs) or processing bodies (P-bodies) in the cytoplasm^[4–5]^. Membrane-less organelles play a significant role in the spatiotemporal regulation of cellular physiological functions, and their dysregulation has been suggested as a hidden mechanism of tumorigenesis (***Fig. 1***).

Phase separation is observed during several normal physiological processes within cells, such as the maintenance of genomic stability and transcriptional activation of target genes, where the phase-separated compartments function as macromolecular reservoirs. Here, we mainly focus on the transcriptional condensates. In brief, the formation of super-enhancers (SEs) is closely correlated with phase separation. Transcription factors (TFs) interact with mediators or coactivators to undergo LLPS and form condensates that may recruit RNA polymerase Ⅱ (Pol Ⅱ). This results in a region of high transcriptional element density that strongly promotes the activation of target genes. The formation of TF condensates on oncogenes may promote the transcription of these oncogenes.

Thus, the present review focuses on the roles of phase separation in transcriptional regulation and how it works in the context of cancer. In addition, we summarize the findings of the recent experiments that have targeted phase separation in cancer treatment. These findings have led to a deeper understanding of cancer development, uncovered novel treatment avenues, and provided a new direction for future investigations.

## From phase separation to condensates

### Mechanisms of phase separation

Recent studies have gradually shifted from investigating the occurrence of phase separation to exploring its internal mechanism. It is now generally accepted that the basis of phase separation is an interaction network built by multivalent protein molecules or RNAs. These multivalent interactions are mediated by multi-folded three-dimensional (3D) regions, intrinsically disordered regions (IDRs), or oligomerized structural domains, among which the most prominent are IDRs^[6]^. IDRs lack a fixed 3D structure but usually contain specific amino acid groups, such as proline, serine, glutamic, tyrosine, and glutamine. The IDRs containing only a few amino acids are known as low-complexity domains (LCDs)^[7]^. A subset of LCDs containing polar and uncharged amino acid residues (*e.g.*, glutamine, asparagine, tyrosine, or serine), which are structurally similar to the yeast prion domain, are called prion-like domains (PLDs). PLDs are often found in RNA-binding proteins (RBPs) and act as drivers of protein phase separation^[8]^. Short amino acid motifs, called "stickers", mediate weak multivalent interactions, including π-π, cation-π, and electrostatic interactions^[9–10]^. TFs serve as critical intracellular regulatory proteins. A recent study demonstrated that the *trans*-activating domains of TFs form condensates through dynamic, multivalent, and specific LCD-LCD interactions that recruit other transcriptional elements^[11]^. In addition to proteins, studies examining the roles of RNA in phase separation have been undertaken in recent years. Typically, RNA interacts with PLD-containing RBPs to induce phase separation. Similarly, Yamazaki *et al*^[12]^ recently found that the long non-coding RNA (lncRNA) *NEAT1* might interact with the RBP non-POU domain-containing octamer-binding through a specific structural domain to undergo phase separation.

By studying intrinsic membrane-less organelles, such as PML bodies and P-bodies, Banani *et al*^[13]^ proposed a "scaffold-client" protein model to elucidate the molecular mechanisms of phase separation. Multivalent scaffold proteins are required for phase separation and may self-organize through heterotypic interactions to drive phase separation. Low-valent client proteins are recruited by interacting and binding to scaffold protein sites, thereby participating in the formation of cell bodies. This recruitment is regulated by the stoichiometric ratio of scaffold proteins, and the valence change of scaffold and client proteins may rapidly alter components of the cell bodies. These findings demonstrate the complexity of intracellular condensates. For example, some cell bodies contain sub-compartments composed of different scaffolds and client proteins.

### Regulation of phase separation

Accumulating evidence suggests that LLPS and condensate formation may be regulated by various factors, whereas the molecular affinity among macromolecules is regulated by physical factors, such as pH, temperature, osmotic pressure, and concentration, which may affect the phase separation process in living cells^[14]^. Because the macromolecules involved in phase separation are mainly proteins, post-translational modifications (PTMs) inevitably affect phase separation by altering the chemical modifications of proteins. When the IDRs of proteins serve as target sites for PTMs that alter their secondary or tertiary structures, the location of their interactions with the IDR also changes^[15]^. Major PTMs that may affect LLPS include phosphorylation, acetylation, methylation, SUMOylation, and ubiquitination. The fused in sarcoma (FUS) protein is a hallmark of neurodegenerative diseases, such as amyotrophic lateral sclerosis (ALS) and frontotemporal dementia (FTD)^[16]^, in which phosphorylation of FUS impairs its aggregation tendency and prion-like characteristics. Although the phosphorylation preserves the inherently disordered region of FUS, transient structural domain collapse, and self-interaction are reduced, and the N-terminus of FUS contains a serine/tyrosine/ glycine/glutamine-rich LCD that acts as a phosphorylation site to regulate condensate assembly^[17]^. Along similar lines, the repetitive FG and RG motifs in probable ATP-dependent RNA helicase DDX4 may be asymmetrically dimethylated, thereby reducing its tendency to undergo LLPS^[18]^. In contrast, myristoylation promotes phase separation, which is a lipid modification process that covalently adds 14-carbon-saturated myristic acid to the amine group of the N-terminal glycine of different cellular proteins; in addition, myristoylation regulates protein-protein or protein-lipid interactions, controls protein stability, and influences transcription^[19]^. Importantly, it drives the homodimerization or heterodimerization of the modified protein through hydrophobic interactions, forming the basis for phase separation. The enhancer of zeste homolog 2 (EZH2) protein is a lysine methyltransferase that is commonly overexpressed in lung cancer cells and exerts oncogenic effects *via* its methyltransferase activity. Investigators have found that myristoylation of a glycine residue at the N-terminus of EZH2 mediates its phase separation and enables the signal transducer and activator of transcription 3 (STAT3) recruitment, and that the enhanced EZH2-STAT3 interaction increases the activation and transcriptional activity of STAT3 and its downstream effectors, ultimately leading to the accelerated proliferation of lung cancer cells^[20]^. This suggests that protein modifications may affect hydrophobic or charged interactions of the original molecule. In addition, some novel modifications, such as palmitoylation, crotonylation, and lactylation, have been relatively less studied, which are important components for future studies on the regulation of phase separation.

Post-transcriptional modifications are also known to regulate LLPS. N6-methyladenosine (m6A) is the most common post-transcriptional modification and has been reported to be correlated with a range of biological activities and diseases^[21]^. YTH N6-methyladenosine RNA binding protein 1 (YTHDF1), YTHDF2, and YTHDF3 are common m6A-binding proteins that may phase-separate *in vitro* and *in vivo*. This ability may be enhanced by mRNAs containing multiple m6A residues. These polymethylated mRNAs are thought to act as "scaffolds" for phase separation, recruiting YTHDF proteins, and interacting with their LCDs to cause phase separation, a process regulated by the number and distribution of m6A sites^[22]^. A similar study reported that YTHDF2 had a weak ability to phase separate intracellularly, but this phase separation ability was significantly enhanced by the addition of m6A mRNA, and an identical effect was observed *in vitro*^[23]^.

Additionally, RNA acts as a regulatory element that modulates the phase separation of proteins. For example, Maharana *et al*^[24]^ reported that RNA concentration might influence the phase separation tendency of prion-like RBPs and that low RNA/protein ratios significantly promoted phase separation, whereas the high ratios had the opposite effect. Another similar study introduced a non-equilibrium RNA feedback control mechanism based on RNA concentration. Low RNA levels promoted the production of condensates formed by electrostatic interactions, whereas relatively high RNA levels promoted the solubilization; during transcription initiation, low levels of short RNAs promoted phase separation; however, once the process was extended, high levels of long RNAs formed by transcription led to condensate lysis^[25]^. In summary, RNAs provide positive and negative feedback by regulating electrostatic interactions in transcriptional condensates, suggesting the fineness and complexity of RNAs in the regulation of cellular biological processes. It is interesting to note that this regulatory effect varies with physiological processes.

### The function of phase separation

Although the functional spectrum of the LLPS-forming condensates has not yet been explored, several key functions have been identified. It has been proposed that intracellular condensates may result from sensing changes in the surrounding environment or stress in response to lethal injury. For example, the poly(A)-binding protein (PABP; Pab1 in budding yeast), an SG marker, has been observed to undergo phase separation under heat stress or altered cytosolic pH^[26]^. In addition, cytosolic acidification caused by cellular energy depletion leads to the phase separation of the budding yeast translation termination factor Sup35^[27]^. The RBP poly(A)-binding protein binding protein 1 (Pbp1 in yeast) senses the redox state of the cell and forms condensates under reducing conditions^[28]^. These examples suggest that phase separation may serve as a tool for maintaining the stability of physiological activities inside and outside the cells. Other studies on genomic and phase separation have led to the gradual recognition of phase separation as an essential means of maintaining genomic stability and chromatin structure. For example, cellular nuclear condensates have been found to act as mechanical chromatin filters that aggregate the distally targeted genomic elements but exclude non-targeted regions of the neighboring genome, demonstrating that nuclear condensates may sense and reconfigure the chromatin environment^[29]^.

The polycomb group proteins may also reorganize the chromatin structure by phase-separated recruitment of DNA and nucleosomes to form expression-silenced parthenogenic chromatin, thereby inhibiting development and differentiation^[30]^. However, condensates are not necessary for maintaining the chromatin-compressed state. In addition to chromatin reconstitution, nuclear condensates often act as "protectors" of the genome. For example, when DNA is fatally damaged by a double-strand break, a functional promoter assembly is induced at this site, including an intact Pol Ⅱ pre-initiation complex, which includes a mediator of Pol Ⅱ transcription subunit 1 (MED1), cyclin-dependent protein kinase 9 (CDK9), and damage-induced lncRNAs; these non-coding RNAs are separated by DNA damage response factors, such as tumor protein p53-binding protein 1, in the form of foci for phase separation to promote DNA repair^[31]^.

In general, when DNA is damaged, global transcription is suspended to maintain transcriptional quality control. Fu *et al*^[32]^ reported that Poly [ADP-ribose] polymerase 1 (PARP1) inactivated positive transcription elongation factor b by multimerization (ADP-ribosylation), disrupting its phase separation, which inhibited Pol Ⅱ elongation and aborted transcription. Furthermore, another study showed that LLPS disruption by using the phase separation inhibitor 1,6-hexanediol caused a slight but irreversible change in the higher-order chromatin structure; specifically, the disruption of weak hydrophobic interactions led to a slight depolymerization and spreading of the nucleosome clusters, partial mixing of the chromatin compartments, changes in the internal structure, and attenuation of the enhancer-promoter loop interactions; all these suggest that phase separation facilitates structural fine-tuning of the 3D genome^[33]^. In short, changes or disturbances in the external environment and internal cell damage are all correlated with phase separation. In most cases, the condensates generated by cellular stress terminate certain biological processes, such as transcription or translation to facilitate self-repair.

Condensates are also believed to act as temporarily isolated sub-compartments that accelerate biochemical reaction kinetics by recruiting specific components to increase their specific activity. For example, the formation of miRNA-induced silencing complex condensates was correlated with the increased deadenylation of RNA^[34]^. Similarly, they may also serve as "storage chambers" for LLPS of proteins or nucleic acids, which is correlated with cell fate transformation and, in turn, is associated with tumorigenesis. For example, serine residues of the heat shock protein HSF1 enable it to undergo LLPS in the form of foci, and the persistence of these foci compromises the protection conferred by the chaperone proteins to the cell, causing apoptosis; conversely, foci degradation promotes HSF1 activity and cell survival; and the physical properties of the foci gradually change to a more static gel-like form with increasing stress periods, suggesting that it may act as a sensor to regulate the cytoprotective response; however, in most malignant cells, HSF1 may lose the ability to undergo LLPS and form foci, thereby inhibiting apoptosis^[35]^. These suggest that condensates are inseparable from the development of various diseases, especially cancer. In conclusion, phase separation is correlated with normal cellular physiological activities, and its inhibition or promotion may lead to abnormal physiological activities.

## Phase separation and transcriptional regulation

### Starting with Pol Ⅱ

Pol Ⅱ plays an important role in the extended phase of transcription in eukaryotic genomes, and its transcriptional regulatory functions are correlated with phase separation. For example, the C-terminal structural domain (CTD) of Pol Ⅱ is a highly conserved sequence of seven amino acid repeat units (Y1S2P3T4S5P6S7) that serves as a regulatory center for transcription and RNA processing and often interacts with transcriptional regulators^[36]^. During transcription, the CTD undergoes dynamic phosphorylation, coordinates the recruitment of other regulatory factors, and tightly couples transcriptional and co-transcriptional RNA processing; specific phosphorylation patterns on the CTD of Pol Ⅱ serve as docking sites for components involved in 5′ mRNA capping, splicing, 3′ end processing, and mRNA export factors^[37–38]^. In summary, the repetitive amino acid structure and chemically modifiable features of the CTD form the basis for the phase separation that occurs when Pol Ⅱ interacts with other regulatory factors. It was reported that CTDs might form condensates and recruit intact Pol Ⅱ through weak multivalent interactions *in vitro* and that the length of the CTD regulated this process, with longer CTDs exhibiting stronger CTD-CTD interactions; additionally, the CTD length was also positively correlated with Pol Ⅱ cluster formation and negatively correlated with droplet kinetics, a phenomenon mediated by hydrophobic interactions that might be abolished by CTD phosphorylation^[39]^. When the CTD length is sufficient to facilitate the recruitment of transcription initiation polymerase to the promoter and delay its release from the promoter, phase separation triggered by the CTD-CTD interactions allows multiple polymerases and transcriptional regulators to condense and interact, leading to a transcriptional burst^[40]^.

**Figure 2:**
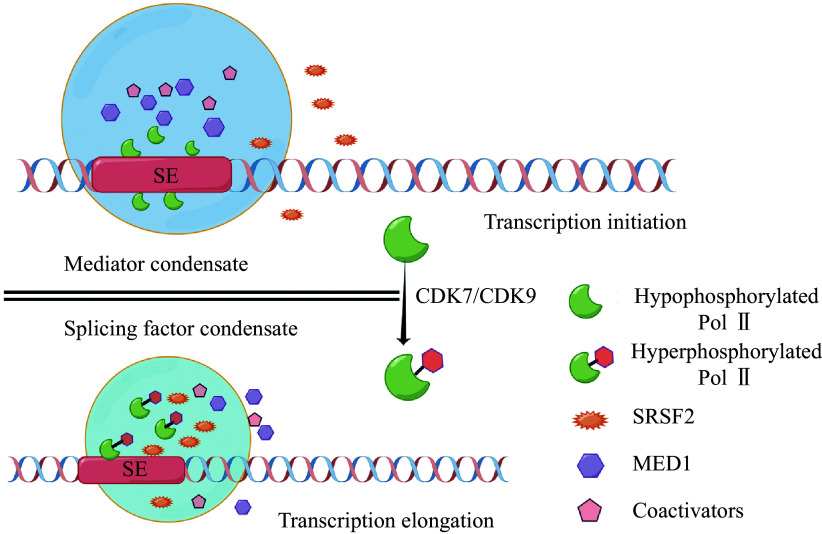
The specific functions of RNA polymerase Ⅱ (Pol Ⅱ) in transcriptional regulation.

Another study focusing on the effect of CTD phosphorylation on phase separation showed that the specific phosphorylation state of the CTD determined whether it interacted with the initiation or splicing complex, and that hypophosphorylated CTDs bound to the mediator condensate, but hyperphosphorylated CTDs predominantly bound to the splicing complex^[41]^. This provides novel insights for studying the specific functions of Pol Ⅱ in transcriptional regulation (***Fig. 2***).

In addition to the intrinsic structure and PTMs of CTDs, other macromolecules are also implicated in CTD-dependent phase separation during transcription. For example, PHD-finger protein 3 (PHF3) docks to the CTD through the SPOC structural domain (considered to be the reading structural domain of the CTD that specifically recognizes the S2 repeat sequence of the phosphorylated CTD) to regulate transcription and mRNA stability^[42]^. PHF3 drives the phase separation of phosphorylated CTDs through hydrophobic interactions, while co-localizing in the Pol Ⅱ cluster to traverse the entire gene length with Pol Ⅱ, a process that is regulated by electrostatic interactions. The entry of PHF3 into the condensate promotes transcriptional elongation and negatively regulates RNA stability^[42]^. Moreover, negative elongation factors undergo dephosphorylation and SUMOylation under stress conditions, forming condensates in the nucleus through the phase separation of IDRs, in which PTMs of negative elongation factors increase their chromatin residence time and inhibit the interaction of Pol Ⅱ with positive elongation factors, thereby downregulating target gene transcription^[43]^.

However, a class of negatively charged short RNAs generated by the random binding of Pol Ⅱ to the chromatin expels the polymerase before the CTD is phosphorylated, which appears to be a negative feedback mechanism. One study used this as the basis for discovering the dynamic and subtle links among RNA, RBPs, and Pol Ⅱ. PSPC1, an RBP, prevented RNA-induced dephosphorylation and CTD expulsion, whereas its LCD interacted with the CTD to phase-separate this region and ensured CTD phosphorylation in the presence of CDK to promote transcription^[44]^. This function is predicated on the LCD and RNA-binding activity of the RBPs.

### In-depth exploration of TFs

TFs are essential for transcriptional regulation. They contain a DNA-binding domain (DBD) and a specific transactivated domain (AD)^[45]^. AD usually contains IDRs composed of low-complexity amino acid sequences, and these IDRs are classified according to their amino acid profiles^[46]^. Hundreds of TFs have been identified in human cells, which may interact with a variety of other transcriptional elements, such as mediators, coactivators, and even Pol Ⅱ, to activate or repress transcription.

OCT4, a major TF required to maintain the pluripotent state of embryonic stem cells, may form droplets with the mediator MED1-IDR at the SE, a process that depends on electrostatic interactions between MED1-IDRs and acidic amino acid residues in the AD of OCT4^[47]^. One subsequent study built on this finding found that neither the glutamine-rich IDR of MED15 nor its hydrophobic amino acid motif was sufficient for condensation; however, the synergistic interaction between these two regions might lead to the onset of phase separation. The CTD of MED15 appears to drive phase separation more efficiently than its IDR^[48]^. This suggests that phase separation is most likely not initiated by a single structural domain, but is the result of the combined actions of several LCDs. The TF-mediated transcriptional condensation is not the only mechanism that promotes transcription. Interactions between ADs may also enhance transcriptional activation by increasing the residence time of the TF chromatin-bound state and facilitating the recruitment of co-activators independent of phase separation.

In addition to classical TFs, hormone receptors, such as the estrogen receptor, androgen receptor, and glucocorticoid receptor, have intrinsic transcriptional activities. They contain ligand-binding domains (LBDs) in addition to DBDs and ADs. For example, one study showed that the glucocorticoid receptor might recruit coactivators through its N-terminal AD, where LLPS condensates occurred, and that the dynamic, multivalent, specific, and stable interactions between the LxxLL motif and the LBD of the glucocorticoid receptor determined the extent of recruitment^[49]^. In addition, DNA binding coordinates the interaction between the disordered N-terminus domain and the folded LBD, which may remove sequences around the interaction site from the pool of amino acids available for dynamic interactions, resulting in condensate formation and compositional bias. Furthermore, it was previously found that the estrogen receptor might form droplets with MED1-IDR *in vitro*^[46]^. The androgen receptor undergoes phase separation through its DBD and AD, and abnormalities in its phase separation functions are strongly associated with prostate carcinogenesis^[50]^.

However, the basis of the 3D spatial structure of the genome formed by transcriptional condensates is poorly understood. Specifically, the 3D chromatin structure is correlated with gene regulation. Topologically associated structural domains form the structural basis of chromatin organization and are characterized by preferential interactions between the chromatin located therein and distinct boundaries, usually forming loops^[51–52]^. The CCCTC-binding factor (CTCF), a TF with 11 zinc finger structural domains, promotes the formation of chromatin loops related to the adhesin complex and delineates the topologically associating domains^[53–54]^. One study has shown that the CTCF-mediated formation of chromatin loop structures is a prerequisite for transcriptional condensate assembly, which provides a spatial framework that facilitates the spatial proximity of transcriptional elements, enhances promoter-enhancer interactions, and promotes transcriptional condensate assembly. The assembly of transcriptional condensates and the formation of chromatin loops are independent events that do not affect each other^[55]^. This finding suggests that the functions of transcriptional condensates are not limited to activating or repressing transcription but involve multiple aspects of the 3D genome.

In summary, the LLPS of TFs is critical for transcriptional regulation of target genes. Initially, a TF binds to its consensus binding sequence in the gene promoter, subsequently recruiting coactivators or mediators to form condensates. The concentration of transcriptional elements greatly increases within this droplet, thus promoting target gene transcription. However, whether this function affects the histones in the LLPS region remains unknown. Because the CTD of Pol Ⅱ is often involved in this process, we speculate that the composition of the transcriptional condensate changes constantly as transcription progresses. The molecules within the condensates may recruit different elements, from the upstream enhancer to the promoter to the downstream coding region, to perform different functions.

### SEs and phase separation are inseparable

The term "super-enhancer" was originally used by Lee *et al* in 2013 to describe a 651-base fragment of baculovirus genomic DNA^[56]^. They observed that this regulatory structural domain increased the activity of the reporter gene promoter by approximately 7000-fold in transgenic cells. Subsequently, ChIP-seq data showed that the SE was enriched with an extremely high density of major TFs and mediators, a structure that plays an important role in transcriptional regulation and disease onset^[57]^. Compared with typical enhancers of 1–4 kb, the average size of SEs is approximately 10–60 kb, a difference of approximately one order of magnitude^[58]^. The formation mechanism of SEs is not yet conclusive, but some studies have suggested that SEs may function as hubs for the accumulation of major TFs and may accelerate the formation of transcriptional condensates. They found that DNA sequences containing TF binding sites with numbers, densities, and affinities above strict thresholds might drive the phase separation of TFs and coactivators. Furthermore, this DNA-dependent effect is particularly evident at low protein concentrations.

In addition, specific TF-DNA interactions may localize TF to the targeted genomic loci, and TF-coactivator interactions may promote transcriptional condensation^[59]^. This finding contributes to the understanding of the link between SEs and transcriptional condensates. Regarding the functional aspects of SEs, no uniform answer has been reached; some believe that SEs are the main regulatory components of gene expression that shape cellular identity, whereas others hold a different view that SEs are nothing more than clusters of enhancers that act additively on their target genes in the form of seat control regions^[60]^.

The most impressive function of SEs is the activation of gene transcription through long-distance chromatin loops that interact with promoters. These structures are regulated and stabilized by a series of *cis*-regulatory elements, and trans-acting proteins are co-localized in each chromatin region^[61]^. TFs have been found to bind tightly to and act synergistically with enhancers to regulate transcriptional activity. One study has pointed out that TFs in enhancer clusters may recruit mediators and interact with Pol Ⅱ to regulate transcription^[62]^. SEs are closely linked to enhancer-TF interactions and are inevitably associated with transcriptional regulation. The transcriptional regulator BRD4, a member of the Bromodomain and extra-terminal domain (BET) protein family, aggregates with MED1 to form puncta in the nucleus as revealed by immunofluorescence analysis. ChIP-seq has suggested that SEs are enriched in BRD4/MED1; therefore, investigators have used DNA/RNA fluorescence *in situ* hybridization to demonstrate the association between BRD4/MED1 and SEs^[63]^. Therefore, MED1 IDR mediates the formation of transcriptional condensates in SEs. Another study on BET proteins found that BET protein inhibitors had a strong effect on transcription but little effect on enhancer-promoter interactions, suggesting that the activation of transcription and enhancer-promoter interactions in the context of SEs are independent events and that CTCF and adhesins are necessary for the latter^[64]^. Previously, we mentioned that CTCF was important for shaping and maintaining the spatial structure of the 3D genome and that it mediated the formation of chromatin loops, which is a spatial prerequisite for the formation of transcriptional condensates. Han *et al*^[65]^ observed that the short isoform of BRD4 (BRD4S), rather than the long isoform (BRD4L), mediated the onset of phase separation, with the latter functioning more towards the recruitment of coactivators and other transcriptional elements into the condensate.

In the context of enhancers and SEs, LLPS plays a critical role in the formation and function of these regulatory elements. TFs and coactivators, which are essential components of enhancers and SEs, have been observed to undergo phase separation. This phase separation may lead to the formation of dynamic and the concentrated hubs of transcriptional machinery at specific genomic loci. For SEs, which involve a dense cluster of TFs and a high level of mediator complex, LLPS may facilitate the formation of these large, complex structures. By concentrating the necessary proteins and regulatory elements in a specific spatial domain, LLPS may enhance the efficiency of transcription initiation and elongation at the target genes. This is particularly important for SEs, as they regulate genes that are crucial for cell identity and function. Furthermore, the dynamic nature of biomolecular condensates formed by LLPS allows for rapid and responsive changes in gene expression. This is vital in cellular processes where timely gene regulation is crucial, such as development, differentiation, and response to environmental stimuli.

In summary, LLPS contributes to the organization and function of enhancers and SEs by facilitating the concentration and co-localization of transcriptional machinery. This enhances the efficiency of transcriptional regulation and plays a crucial role in the dynamic control of gene expression. Understanding this interplay is important for comprehending how cells precisely regulate gene expression in various biological contexts and diseases.

### Phase separation also participates in post-transcriptional modifications

It is well understood that to perform their physiological functions, pre-mRNAs undergo a series of post-transcriptional modifications after transcriptional formation, such as the attachment of 7-methyl guanosine at the 5′ end, the formation of a polyadenylated tail at the 3′ end, and structural splicing^[66]^. Of the many types of mRNA modifications in mammals, m6A modifications are the most abundant^[67]^.

Investigators have found that m6A plays an important role in a variety of biological processes, including RNA nuclear export, mRNA splicing, miRNA processing, mRNA degradation, and translation^[68–69]^. As mentioned before, m6A modifications may be recognized by the m6A reading proteins, such as the very specific YTHDF1/2/3, where YTHDF1 and YTHDF3 facilitate the translation of m6A mRNAs, while YTHDF2 and YTHDF3 mediate the degradation of m6A mRNAs^[70]^. The C-terminus of this protein family contains an LCD that allows it to undergo phase separation^[71]^. However, a recent study found that YTHDF1 could also degrade target mRNAs by recruiting AGO2 proteins through the YTH structural domain to undergo phase separation, facilitating the formation of P-bodies for mRNA degradation^[72]^. Coincidentally, the AGO protein family is an important component of RNA-induced silencing complex (RISC) that exerts post-transcriptional repression by recognizing target mRNAs and binding to their 3′ or 5′ ends.

One study has found that the miRNA-induced silencing complex formed by miRNA binding to RISC may repress CDK1 in an LLPS-dependent manner^[73]^. Lee *et al*^[74]^ have found that m6A-enhancer RNA labels highly active enhancers and recruits the reading protein YTHDC1, which acts through its C-terminal IDR and arginine residues to promote LLPS and activate transcription. To their surprise, the formation of the YTHDC1 condensate promoted the formation of and comixing with the acetylated histone reader BRD4 condensate. This phenomenon reveals a crosstalk between the epigenome and epi-transcriptome, which should be explored in depth in the future.

## Involvement of phase separation in tumorigenesis

### Oncogenic fusion proteins formed by phase separation

The intersection of LLPS and fusion proteins is an emerging area of interest, particularly in understanding how phase separation may influence the behavior and pathogenicity of fusion proteins. For example, the altered physical properties of a fusion protein may impact its propensity to undergo LLPS, potentially leading to the formation of pathological aggregates, as observed in some neurodegenerative diseases. Additionally, fusion proteins may aberrantly participate in or disrupt normal LLPS processes, affecting the formation and function of membrane-less organelles, and thereby impacting cellular homeostasis and functions. Ewing sarcoma breakpoint region 1-friend leukemia integration 1 (EWS-FLI1) is an oncogenic TF that plays a key role in the development of Ewing sarcoma. This fusion results from the PLD of FET family proteins (FUS, EWS RNA binding protein 1 [EWSR1], and TATA-box binding protein (TBP)-associated factor 15 [TAF15]) with the DBD of the TF^[75–76]^.

Recent studies have also revealed that FET fusion proteins may form condensates at the DBD-binding site of the fusion TF and recruit the CTD of Pol Ⅱ to the binding motif, thereby promoting oncogene transcription, a process that relies on both LCD-LCD and LCD-DBD interactions. In addition, the formation of FET fusion protein condensates revolves around DNA-binding motifs and has a numerical threshold. Once a specific target motif (total motif number > threshold) is present, the site tends to be enriched for FET fusion proteins, and phase separation occurs at a certain concentration, which leads to transcriptional activation of the target gene^[77]^. Another study on EWS-FLI1 has revealed that this fusion TF requires fine-tuned LCD-LCD interactions to activate the target gene with a narrow range of adaptation levels. When EWS-FLI1 is recruited to the nucleolus, it may overcome the binding interaction with DNA, and ectopic LCD-LCD interactions segregate them into phase-separated subunits^[78]^, in which the transcriptional activity of EWS-FLI1 is inhibited by the ectopic LCD-LCD interaction. This finding suggests that TFs require fine-tuned optimal levels of LCD-LCD interactions to activate transcription. This reveals an important property of the LCD self-interaction in EWS that could be a potential new therapeutic opportunity.

The FUS is a protein that plays a significant role in various cellular processes, such as RNA processing and DNA repair. As a member of the FET family of RBPs, FUS is known for its involvement in the development of certain neurodegenerative diseases and cancers. Similar to EWSR1 in Ewing's sarcoma, FUS may be involved in chromosomal translocations, leading to the formation of fusion proteins^[79]^. These fusion proteins may act as aberrant TFs or disrupt other cellular processes, contributing to the development and progression of cancer.

Importantly, FUS is one of the proteins known to undergo LLPS. This property allows FUS to form dynamic, membrane-less organelles in the cells, such as SGs. LLPS is crucial for the normal function of FUS in RNA metabolism and cellular stress responses. However, in pathological conditions, such as certain mutations correlated with amyotrophic lateral sclerosis or frontotemporal dementia, this phase separation behavior may become dysregulated, leading to the formation of persistent and aberrant aggregates that are toxic to cells. Thus, FUS is a key protein in understanding the intersection of RNA biology, neurodegeneration, and cancer biology, and its behavior in LLPS provides significant insights into these diverse medical conditions.

In addition to activating oncogene transcription, these fusion-forming oncoproteins have been reported to affect chromatin remodeling. For example, the FET fusion protein FUS-DDIT3 undergoes PLD-mediated phase separation and forms a condensate that recruits transcriptional elements, such as chromatin remodelers switch/sucrose non-fermentable, mediating both chromatin remodeling and oncogenic transcriptional events^[80]^. Condensates formed by these fusion proteins often co-localize with other functional elements. For instance, the mimicry switch (ISWI) family members, SMARCA5 and BPTF, are found in the condensates of NUP98-NSD1, which are common in childhood acute myeloid leukemia. In addition, NUP98 interacts with the transcriptional coactivators EP300 and KMT2A^[81]^. A more specific interaction occurs in renal cell carcinoma with the NONO-TFE3 translocation, where the NONO fragment may maintain the stability of the fusion protein by phase separation to inhibit its degradation, and another TF, NRF1, may form a positive feedback loop with NONO-TFE3 to promote the transcriptional activity of the latter^[82]^. In conclusion, aberrantly fused oncoproteins may recruit other elements to activate oncogene transcription by phase separation of some of their fragments; however, this action may only occur under certain conditions.

### TF condensates promote cancer development

**Figure 3:**
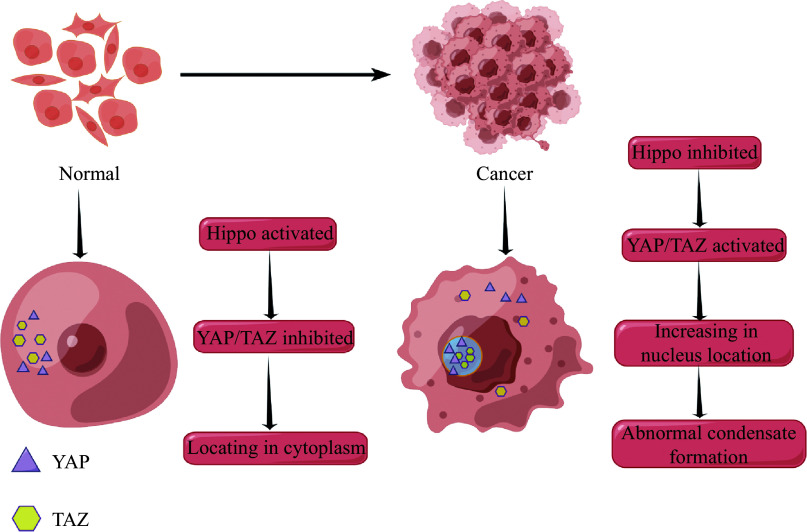
Activation of the YAP/TAZ in cancer tissues.

It has been previously mentioned that major TFs form condensates with other transcriptional elements, such as coactivators and mediators, at SEs to recruit Pol Ⅱ to activate target gene transcription. In tumors, genomic alterations promote the formation of oncogene SEs that activate oncogenic transcriptional programs^[83]^. Thus, the formation of aberrant condensates in SEs may be a general mechanism by which cancer cells maintain high oncogene expression levels. Typical examples are the effectors of the Hippo pathway, the yes-associated protein (YAP), and the transcriptional coactivator with the PDZ-binding motif (TAZ), which regulate cell proliferation, tissue homeostasis, and tumorigenesis. The Hippo-activated LATS1/2 phosphorylates the key transcriptional effectors YAP/TAZ, leading to their increased cytoplasmic localization and transcriptional activity inhibition^[84–85]^. In normal tissues, Hippo is in an activated state; however, in tumors, where normal tissue is disrupted, the Hippo signaling is inhibited, leading to an increased nuclear localization and an aberrant activation of YAP/TAZ, which controls gene expression through direct control of the TF TEAD family^[86]^ (***Fig. 3***). Intracellular YAP forms condensates of the intrinsic TF TEAD1 in the SE region and appears to recruit Pol Ⅱ to trigger transcription of proliferation-related genes^[87]^. TAZ may also undergo phase separation in the nucleus through its coiled-coil structural domain, forming condensates that recruit TEAD4 and the coactivators BRD4, MED1, and CDK9. However, YAP and TAZ are phase-separated under different conditions, possibly because of the differences in their CC structural domains^[88]^. Studies have also observed that the YAP condensate contains some accessible chromatin regions and that Pol Ⅱ, although not within the droplet, later localizes at the edge of the droplet, suggesting that the YAP condensate may dynamically reconfigure the genomic environment to drive the long-term expression of target genes.

In a study on glioblastoma, investigators used a biotinylation approach to search for the TAZ-binding proteins in glioblastoma cells and identified the NONO protein, which was essential for TAZ to form phase-separated condensates in the nucleus and to enhance the interaction between TAZ, TEAD, and Pol Ⅱ subunit B1, ultimately leading to the enhanced transcription of oncogenic genes^[89]^. It has also been observed that NONO proteins may bring TAZ close to the enhancers and promoters of target genes, which provides an idea of the localization mechanism of various transcriptional elements. Tripathi *et al*^[90]^ also observed that YAP and TAZ had a crosstalk with the TGFβ/Smad and Wnt/β-catenin pathways and that Wnt signaling increased the nuclear localization of TAZ and β-catenin; when TAZ activation by the Hippo pathway was inhibited, SMAD7 and β-catenin activity at the myocyte promoter was also reduced, and TAZ exhibited phase-separating properties and recruited β-catenin in myocytes. The authors have proposed a model in which TAZ acts as a balancing fulcrum for myogenic cell proliferation and differentiation, maintaining the undifferentiated and proliferative states of myoblasts. Another TF found to localize to SEs is YY1, which mediates phase separation in the nucleus *via* a histidine cluster in its AD, recruiting coactivators EP300, BRD4, and MED1 to form an enhancer cluster linking target gene promoters, while activating Pol Ⅱ to promote oncogenic gene transcription^[91]^.

## May phase separation be used to explore new treatment options?

Given the increasing understanding of the link between phase separation and tumors, it is logical to consider whether this phenomenon may be used as a target for cancer therapy. Many investigators have explored this issue and produced innovative results. The structural basis for phase separation is the weak multivalent interactions mediated by IDRs containing specific amino acids. Therefore, targeting IDRs may be a possible approach. Investigators took advantage of the fact that the AAA+ protein HSP104 could deconjugate yeast prion-like granules and constructed an enhanced variant of HSP104 to target the prion-like protein regions, which successfully inhibited the translocation of fusion oncoproteins FUS-CHOP and EWSR1-FLI^[92]^. From the perspective of tumor cell characteristics, genetic alterations in cancer cells often lead to dysregulation of the transcriptional program, making cancer cells dependent on certain critical regulators of oncogene expression, a phenomenon known as "transcriptional addiction"^[93]^. This phenomenon is believed to be activated and maintained by SEs. To test this, Lu *et al*^[94]^ used the H3K27me3 demethylase inhibitor GSK-J4 to disrupt transcriptional condensates and showed that the disruption of transcriptional condensates inhibited tumor metastasis and reversed drug resistance. A natural product called PCG (procyanidin C-13,3′,3″-tri-O-gallate; previously called REJ-C1G3) from *Polygonum cuspidatum*, a traditional Chinese medicinal herb, was found to target BRD4. PCG induces misfolding of BRD4 by aggregating the proline sequence in BRD4, inhibiting its phase separation and ultimately making the BRD4 phase separation condensate more stable and static^[95]^. Another novel compound, aminocyclopropenone, may similarly impair the BRD4-driven phase separation by inhibiting oncogene MYC expression and thus inhibit tumor growth^[96]^. These examples suggest novel therapeutic strategies for disrupting the phase separation process to disable downstream effectors.

Phase separation has also been observed in other drug resistance mechanisms that arise during drug therapy. Small-molecule drugs selectively aggregate in certain condensates, even in the absence of a drug target; for example, cisplatin selectively aggregates in MED1 droplets, and mitoxantrone aggregates in MED1, FIB1, and NPM1 droplets. Specifically, in a tamoxifen resistance model, the overexpression of MED1 led to the formation of larger droplets, in which the aggregated tamoxifen was diluted, leading to a reduced efficacy^[97]^. Further investigations are required to determine whether cancer cells develop drug resistance through this mechanism by recruiting small-molecule drugs and altering the nature of the condensate. The above examples suggest that a combination of phase separation-based phenotypic screening and functional analysis is a viable approach to discovering appropriate drugs that target refractory tumors.

## Conclusions and perspectives

LLPS is currently involved in almost all cellular aspects, and investigators worldwide are increasingly studying this phenomenon. Abnormalities in phase separation are also gradually being identified in many diseases, such as neurodegenerative diseases and cancers. A typical example of the latter is the formation of condensates from major TFs, coactivators, and mediators that aberrantly activate the transcription of oncogenes. An increasing number of tumor-associated proteins whose functions are regulated by phase separation have been identified and characterized. Their regulation by phase separation may affect genomic structure, function, and cancer cell life course.

In the present review, we have explained how phase separation exerts regulatory functions in the transcriptional condensates. We have also discussed recent research strategies for treating tumors by targeting phase separation. Recent studies on phase separation have provided valuable insights into the pathophysiological processes of organisms and the mechanisms underlying the development of various diseases. However, phase separation remains a growing research direction, and accurately controlling it *in vivo* and *ex vivo* during the research process remains challenging. Studies on the roles of phase separation in tumor regulation have focused on the functions of condensates formed by phase separation in cancer cells. Furthermore, many studies have been performed to verify whether proteins may be phase-separated by *in vitro* purification. However, the specific roles of phase separation in tumor development and metastasis as well as the relevant mechanisms involved remain to be explored.

Because of the complexity of the intracellular environment and molecular regulatory mechanisms, the *in vitro* conditions cannot completely replace the intracellular environment. Fortunately, in recent years, some investigators have combined optogenetics with phase separation studies, an approach that allows for dynamic observation of protein interactions in living cells, by setting an "optogenetic switch" for spatiotemporal control of intracellular phase separation. Such an approach is likely to provide invaluable insights, compared with traditional *in vitro* protein reconstruction methods. In addition, 1,6-hexanediol is a frequently used phase separation inhibitor that interferes with condensation *in vitro* and *in vivo* by disrupting hydrophobic interactions among proteins. However, this action is often nonspecific and does not meet increasingly precise research needs. Therefore, new and more specific methods for perturbing phase separation are urgently needed.

In conclusion, LLPS is a novel biochemical phenomenon that has greatly contributed to the understanding and study of many diseases. Although the techniques and methods reviewed here remain immature, there is reason to believe that continued research in this area will reveal new concepts and identify novel therapeutic targets, thereby bringing us closer to achieving effective cancer treatment goals.
